# Supplementary material for: Bioactivity-Guided Screening of Antimicrobial Secondary Metabolites from Antarctic Cultivable Fungus Acrostalagmus luteoalbus CH-6 Combined with Molecular Networking
Source: Mar Drugs. 2022 May 19;20(5):334. doi: 10.3390/md20050334 (PMC9146861; doi:10.3390/md20050334)
Supplement: Supplementary file 1 [file marinedrugs-20-00334-s001.zip › marinedrugs-1713083-supplementary.pdf]

Supplementary Material

Bioactivity-guided Screening of Antimicrobial Secondary Metabolites from Antarctic Cultivable Fungus *Acrostalagmus luteoalbus* CH-6 Combined with Molecular Networking

Ting Shi[1,2], Xiang-Qian Li[2,3], Ze-Min Wang[2], Li Zheng[4,5], Yan-Yan Yu[2], Jia-Jia Dai[2] and Da-Yong Shi[2,3*]

[1]College of Chemical and Biological Engineering, Shandong University of Science and Technology, Qingdao, 266590, China

[2]State Key Laboratory of Microbial Technology, Institute of Microbial Technology, Shandong University, Qingdao 266200, China

[3]Laboratory for Marine Drugs and Bioproducts of Qingdao National Laboratory for Marine Science and Technology, Qingdao 266071, China

[4]Key Laboratory of Marine Eco-Environmental Science and Technology, First Institute of Oceanography, Ministry of Natural Resources, Qingdao, 266061, China

[5]Laboratory for Marine Ecology and Environmental Science, Qingdao Pilot National Laboratory for Marine Science and Technology, Qingdao, 266071, China

Figure S1. Fermented cultivable fungal colonies from Fildes Peninsula, Antarctica
Figure S2. The colony morphology (left) and light microscopy (right) of *A. luteoalbus* CH-6
Figure S3. ^{1}H NMR (600 MHz, $CDCl_3$) spectrum of compound **1**
Figure S4. ^{13}C NMR (150 MHz, $CDCl_3$) spectrum of compound **1**
Figure S5. HSQC ($CDCl_3$) spectrum of compound **1**
Figure S6. COSY ($CDCl_3$) spectrum of compound **1**
Figure S7. HMBC ($CDCl_3$) spectrum of compound **1**
Figure S8. NOESY ($CDCl_3$) spectrum of compound **1**
Figure S9. HR-ESI-MS spectrum of compound **1**
Figure S10. ^{1}H NMR (600 MHz, $CDCl_3$) spectrum of compound **2**
Figure S11. ^{13}C NMR (150 MHz, $CDCl_3$) spectrum of compound **2**
Figure S12. HSQC ($CDCl_3$) spectrum of compound **2**
Figure S13. COSY ($CDCl_3$) spectrum of compound **2**
Figure S14. HMBC ($CDCl_3$) spectrum of compound **2**
Figure S15. NOESY ($CDCl_3$) spectrum of compound **2**
Figure S16. HR-ESI-MS spectrum of compound **2**
Figure S17. ^{1}H NMR (600 MHz, $CDCl_3$) spectrum of compound **1s**
Figure S18. HR-APCI-MS spectrum of compound **1s**
Figure S19. ^{1}H NMR (600 MHz, $CDCl_3$) spectrum of compound **1r**
Figure S20. HR-APCI-MS spectrum of compound **1r**
Figure S21. ^{1}H NMR (600 MHz, $CDCl_3$) spectrum of compound **2s**
Figure S22. HR-APCI-MS spectrum of compound **2s**
Figure S23. ^{1}H NMR (600 MHz, $CDCl_3$) spectrum of compound **2r**
Figure S24. HR-APCI-MS spectrum of compound **2r**
Figure S25. ^{1}H NMR (600 MHz, $CDCl_3$) spectrum of compound **3**
Figure S26. HR-ESI-MS spectrum of compound **3**
Figure S27. ^{1}H NMR (600 MHz, $CDCl_3$) spectrum of compound **4**
Figure S28. HR-ESI-MS spectrum of compound **4**
Figure S29. ^{1}H NMR (600 MHz, $CDCl_3$) spectrum of compound **5**
Figure S30. HR-ESI-MS spectrum of compound **5**
Figure S31. ^{1}H NMR (600 MHz, CD_3OD) spectrum of compound **6**
Figure S32. HR-ESI-MS spectrum of compound **6**
Figure S33. HPLC fingerprints of separated fractions Fr.1–Fr.8 of *A. luteoalbus* CH-6
Table S1. Identified compounds by molecular networking
Table S2. OR values of compounds **4**–**6**
Table S3. Antimicrobial activities of the separated fractions (50 μg/mL) of *A. luteoalbus* CH-6

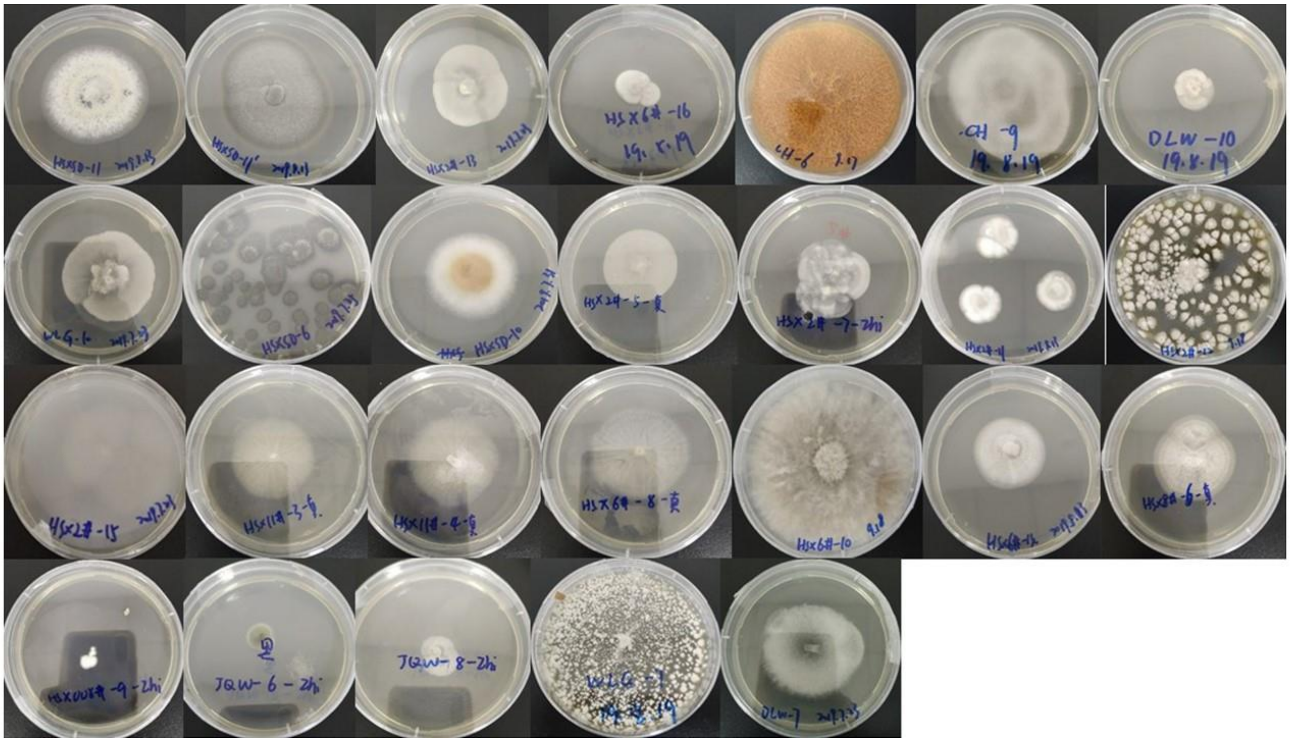

Figure S1. Fermented cultivable fungal colonies from Fildes Peninsula, Antarctica

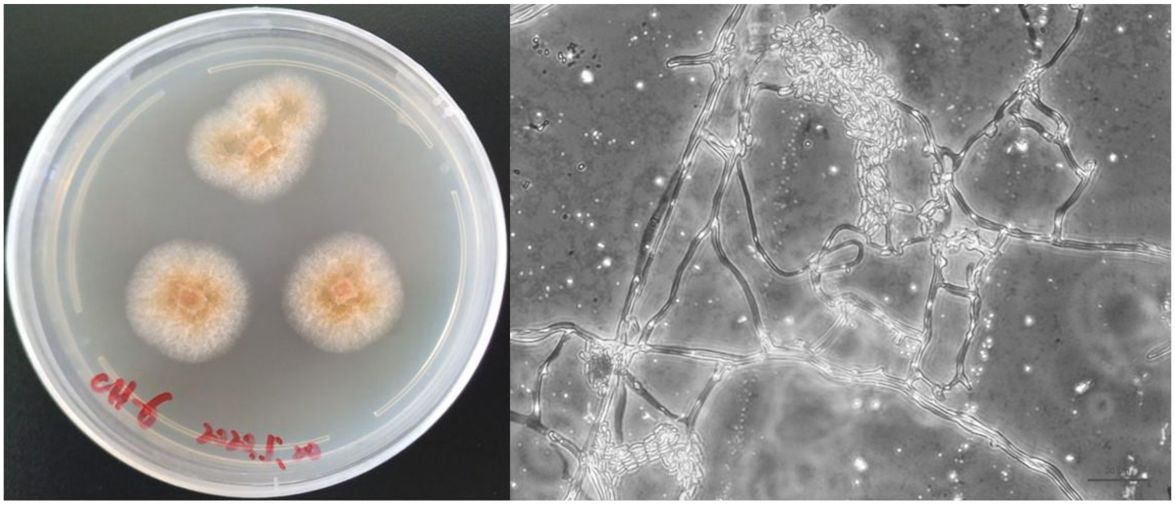

Figure S2. The colony morphology (left) and light microscopy (right) of *A. luteoalbus* CH-6

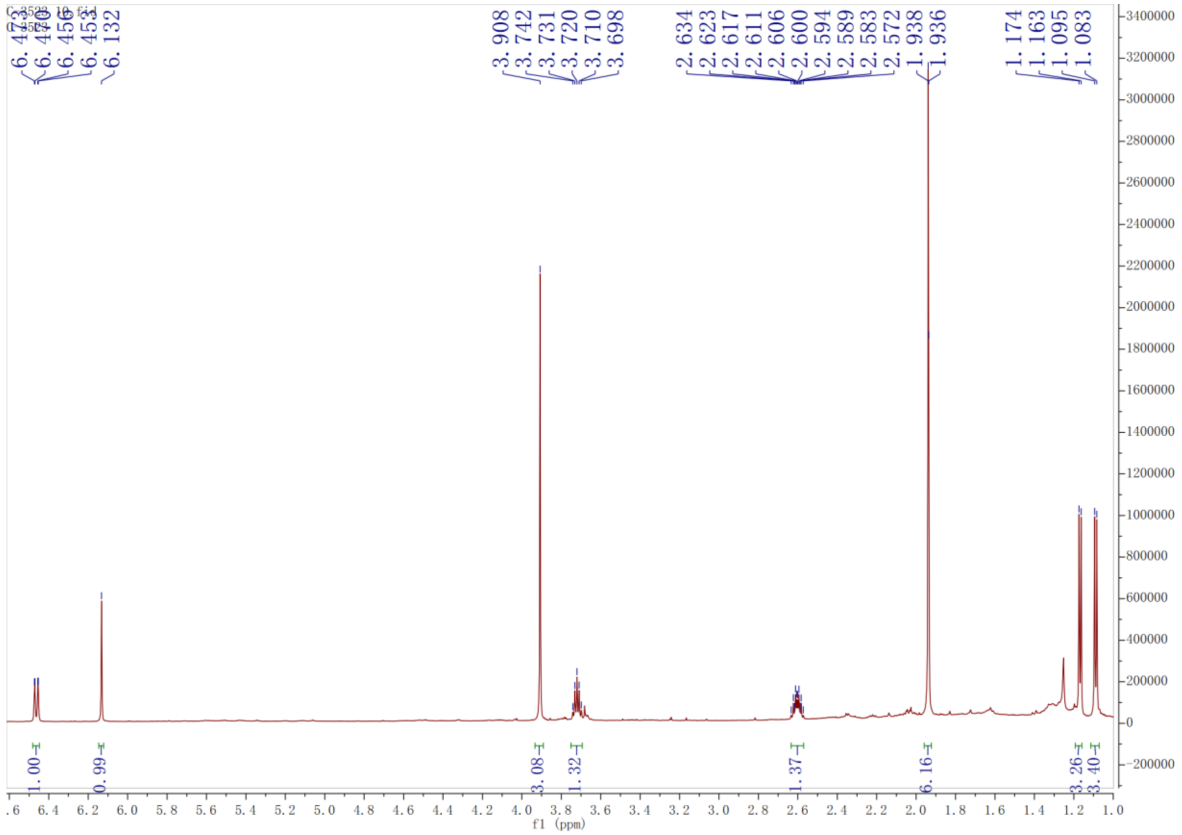

Figure S3. ^{1}H NMR (600 MHz, $CDCl_3$) spectrum of compound **1**

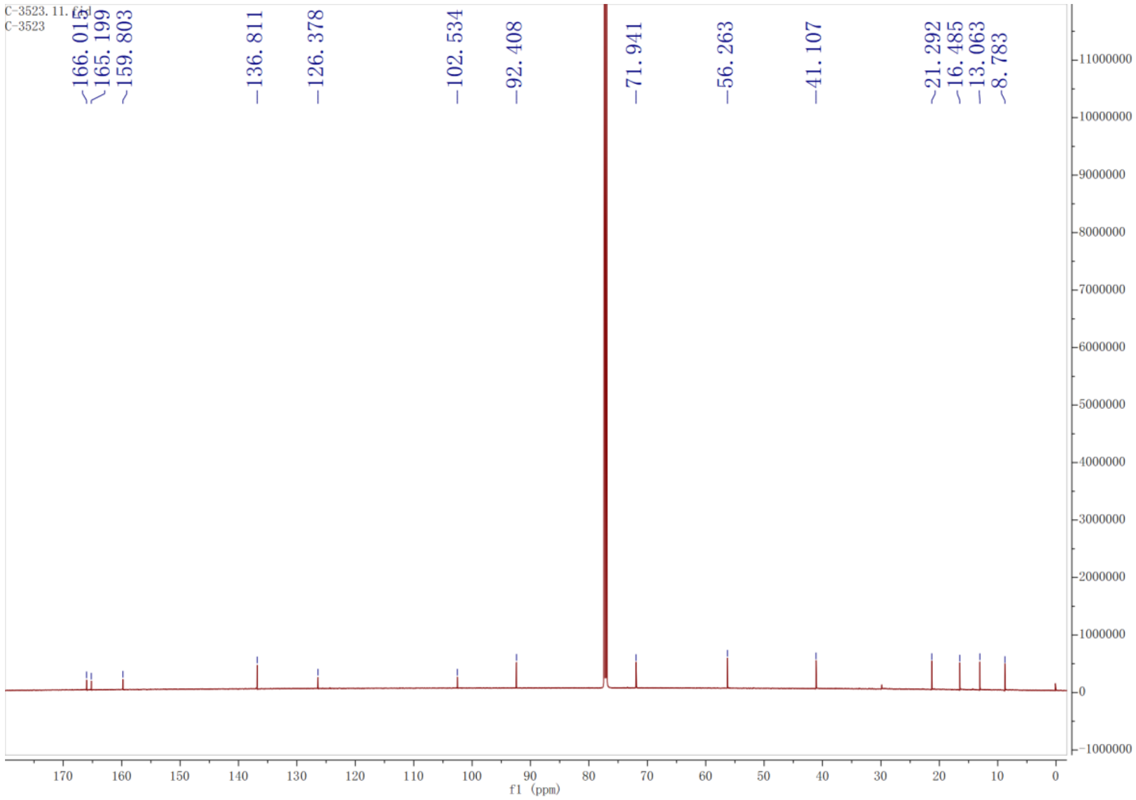

Figure S4. ^{13}C NMR (150 MHz, $CDCl_3$) spectrum of compound **1**

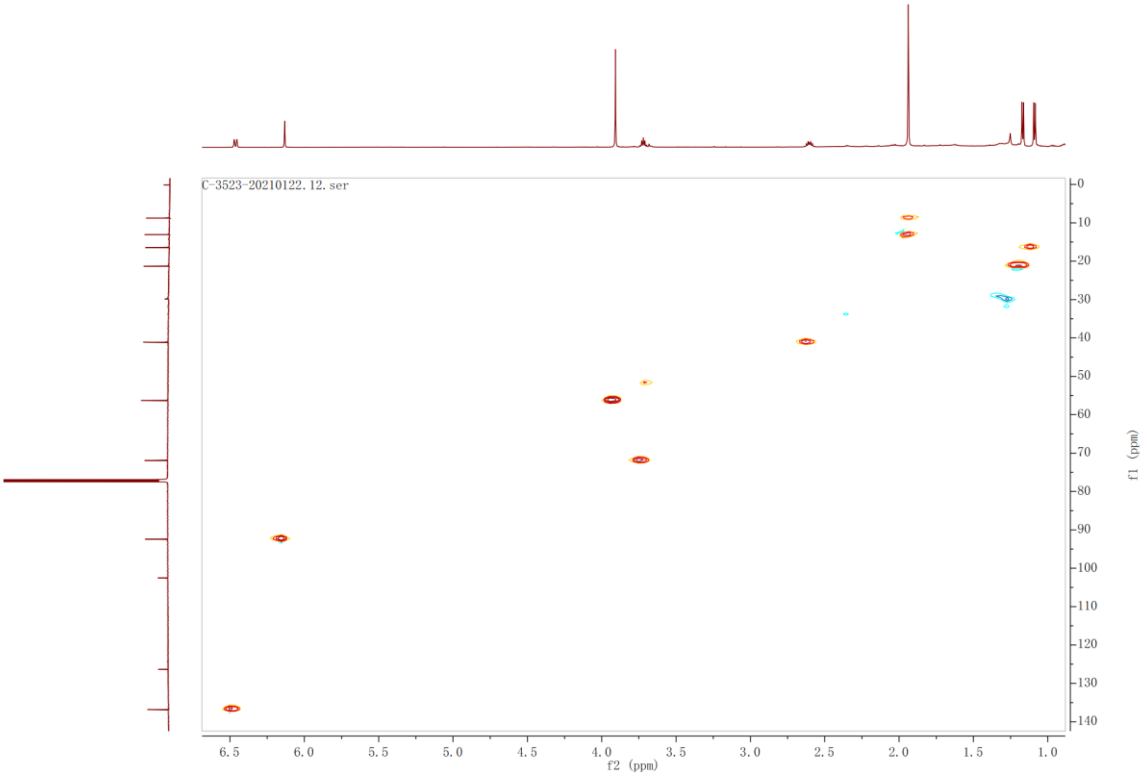

Figure S5. HSQC ($CDCl_3$) spectrum of compound **1**

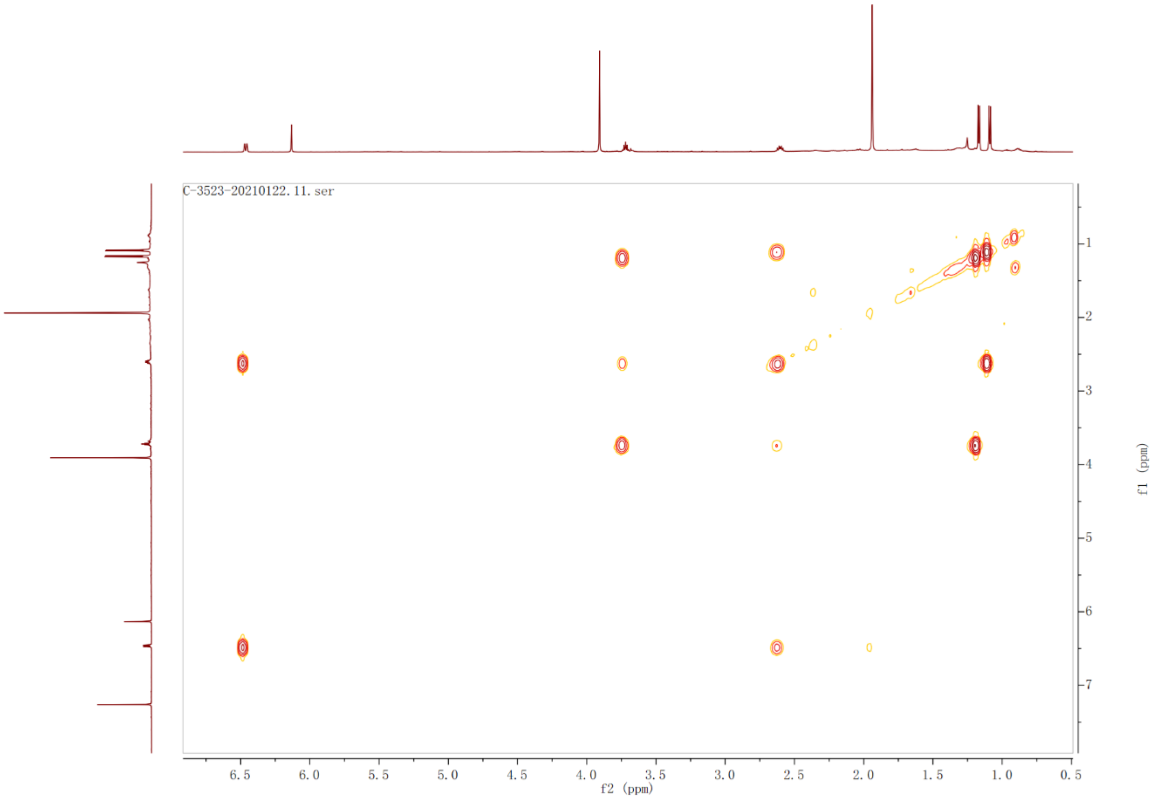

Figure S6. COSY ($CDCl_3$) spectrum of compound **1**

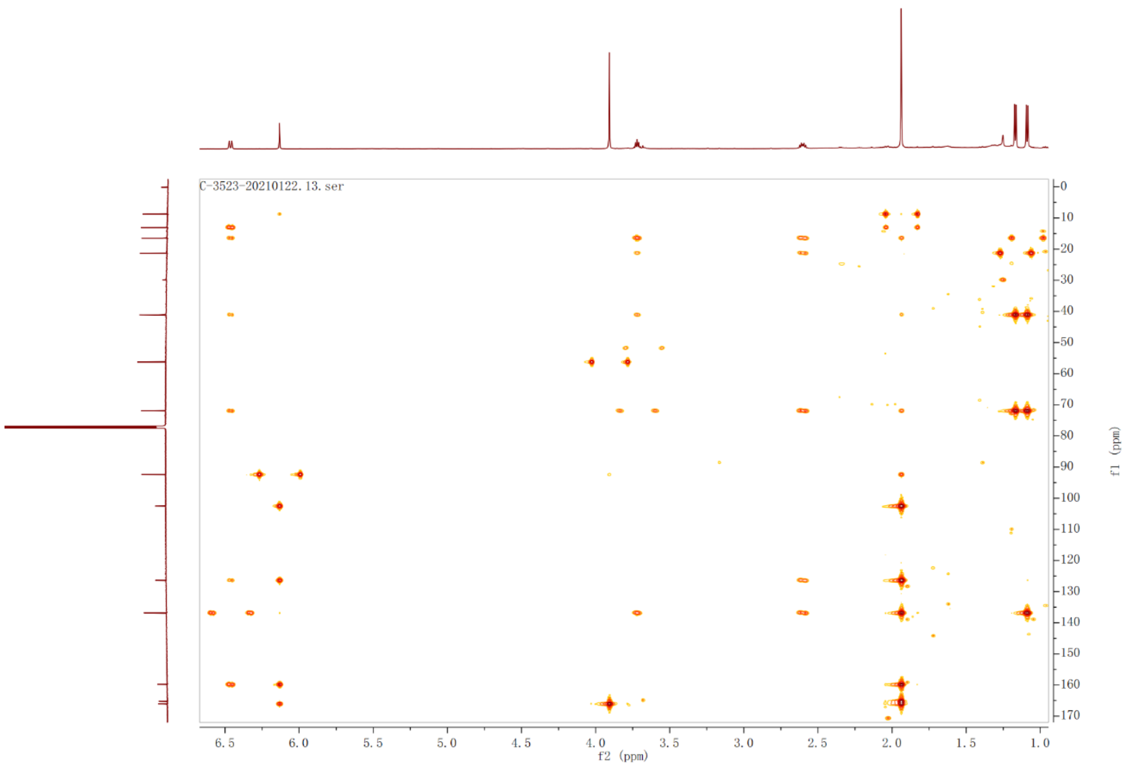

Figure S7. HMBC ($CDCl_3$) spectrum of compound **1**

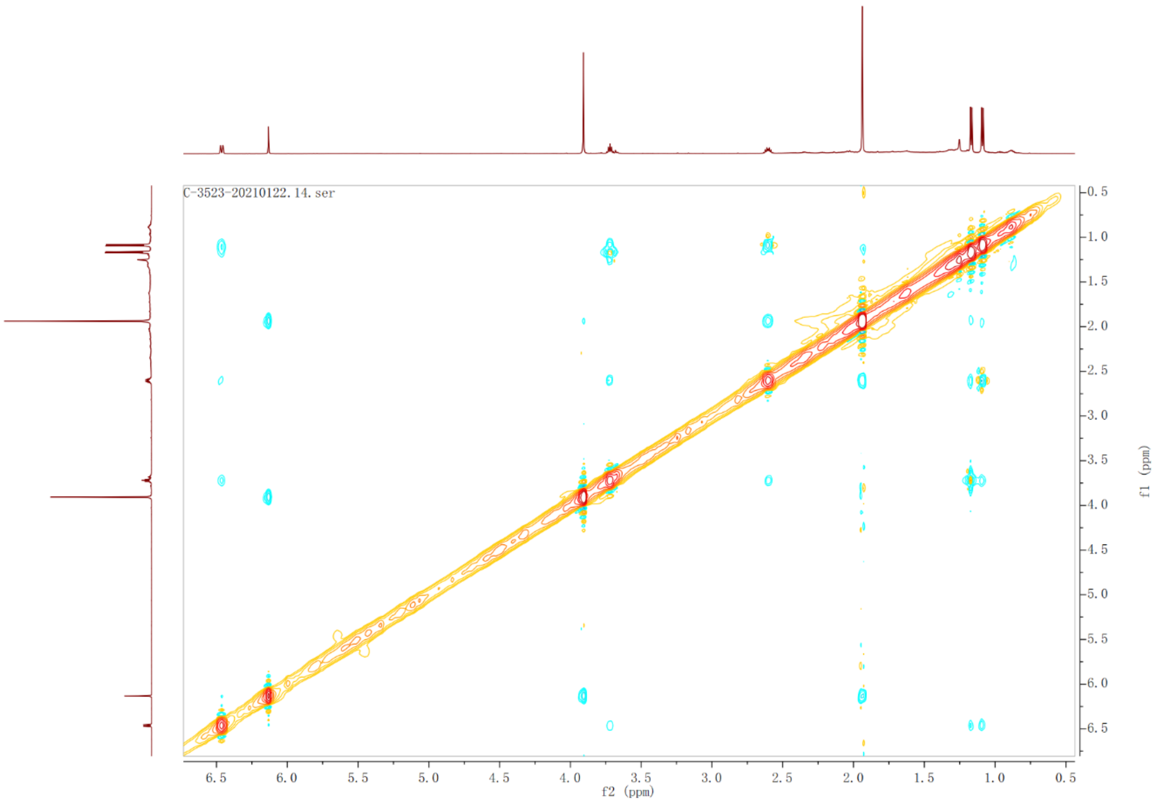

Figure S8. NOESY ($CDCl_3$) spectrum of compound **1**

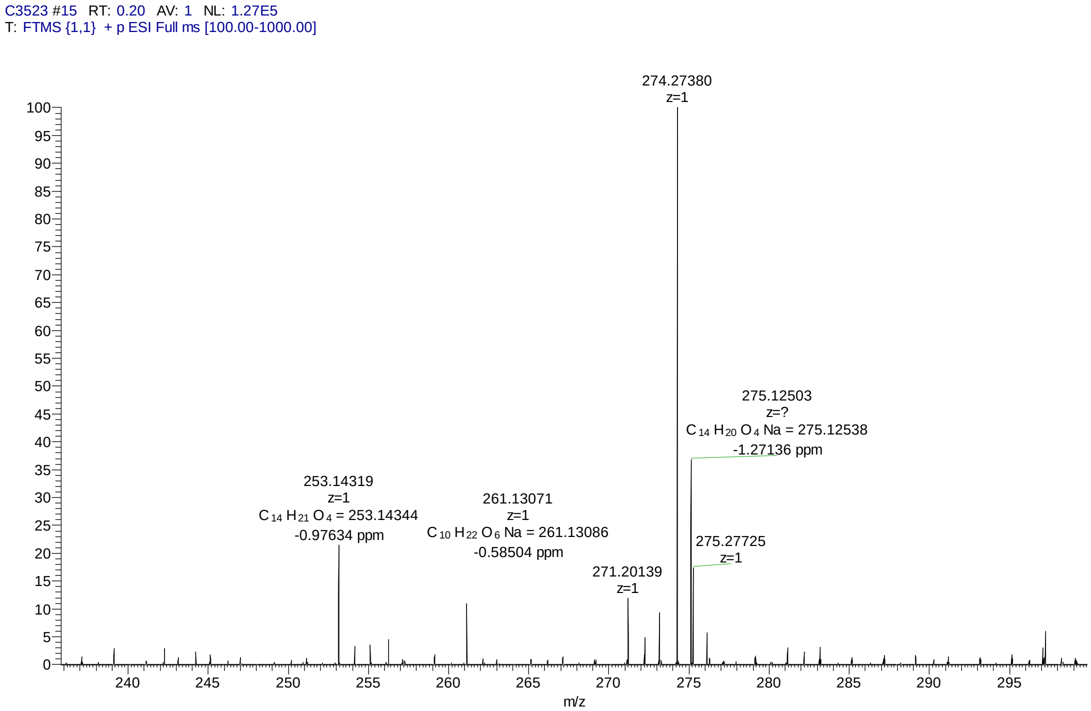

Figure S9. HR-ESI-MS spectrum of compound **1**

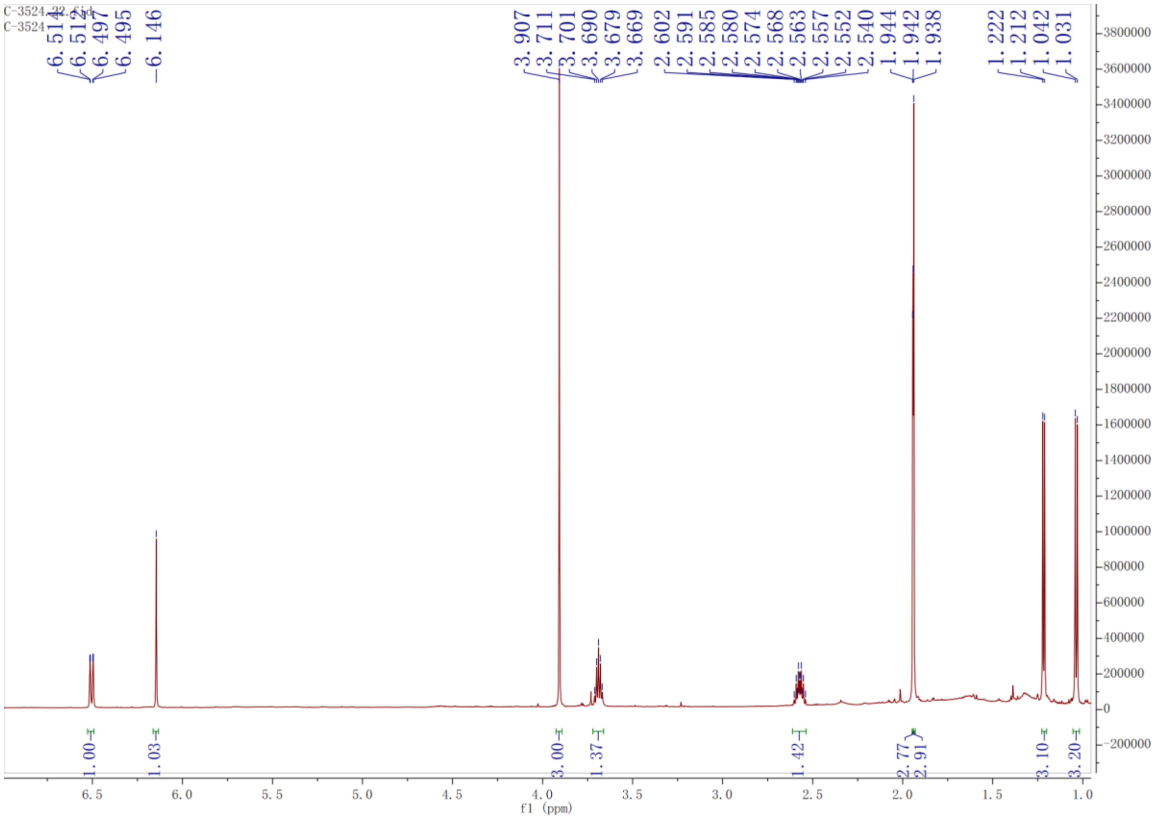

Figure S10. ^{1}H NMR (600 MHz, $CDCl_3$) spectrum of compound **2**

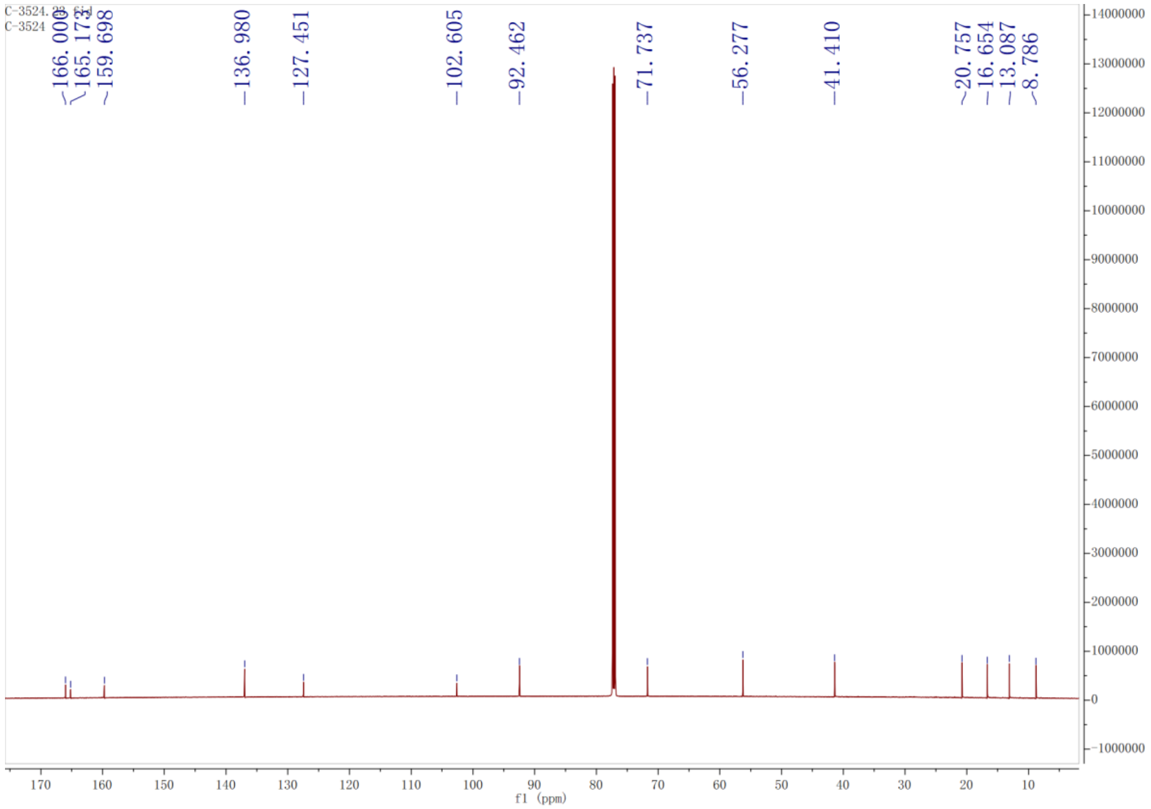

Figure S11. ^{13}C NMR (150 MHz, $CDCl_3$) spectrum of compound **2**

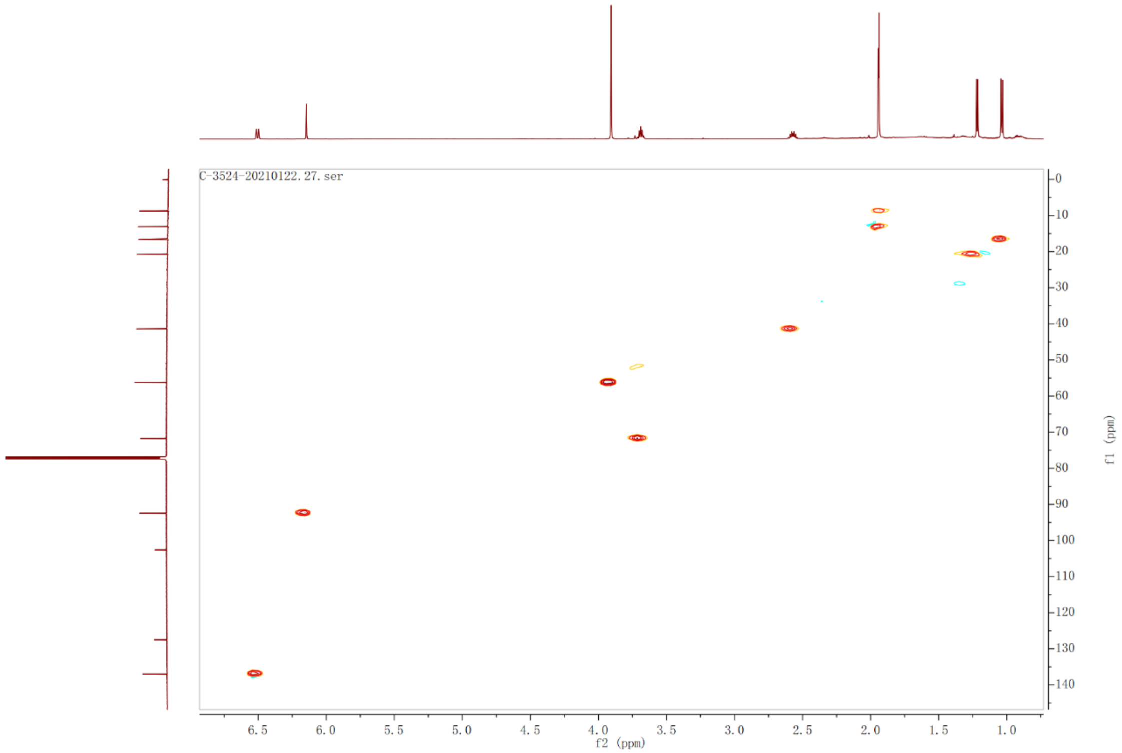

Figure S12. HSQC ($CDCl_3$) spectrum of compound **2**

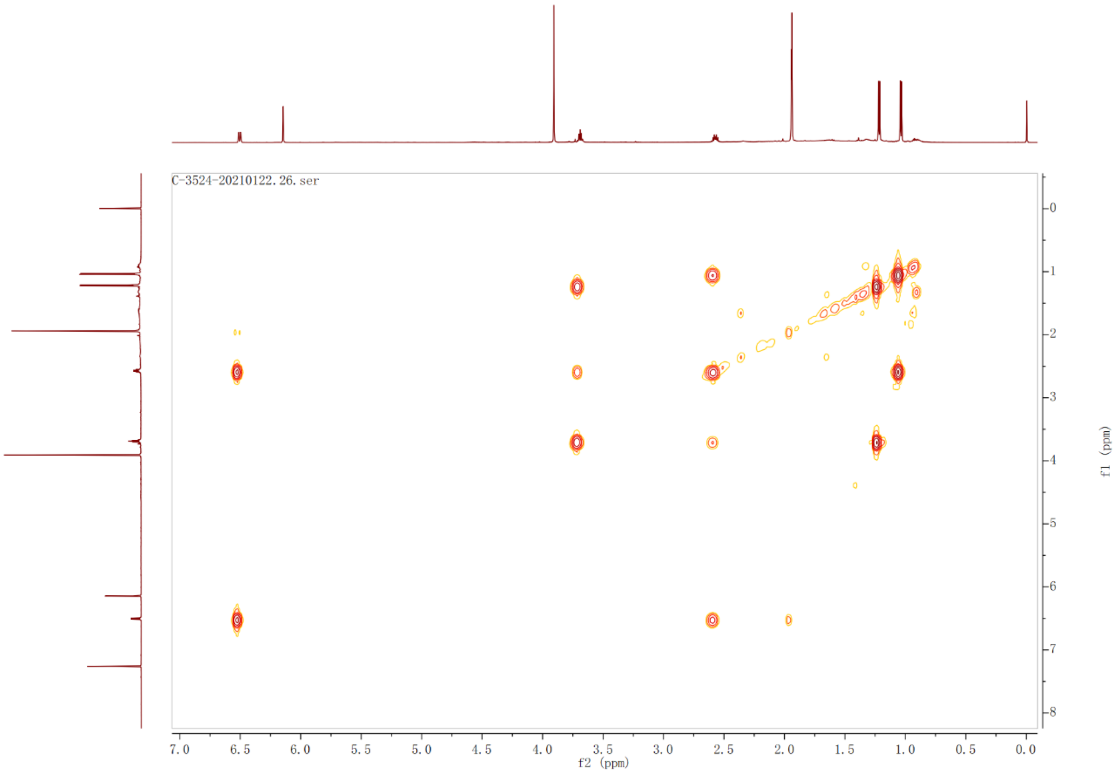

Figure S13. COSY ($CDCl_3$) spectrum of compound **2**

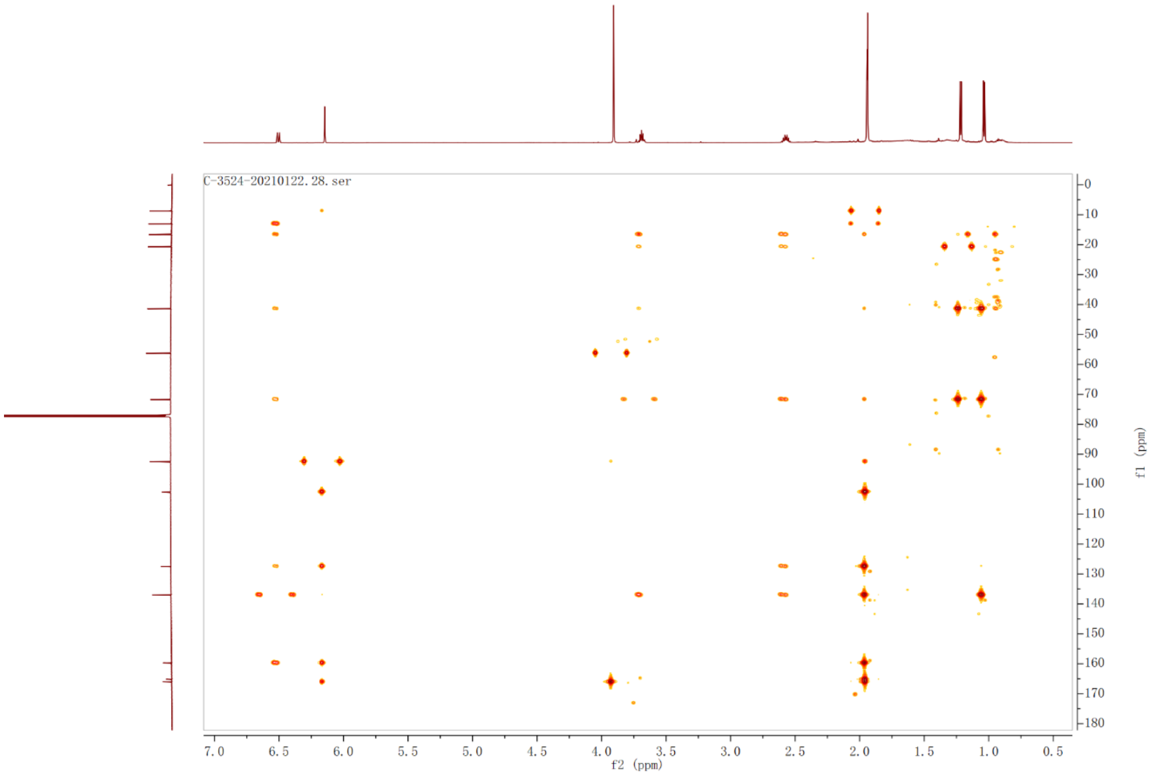

Figure S14. HMBC ($CDCl_3$) spectrum of compound **2**

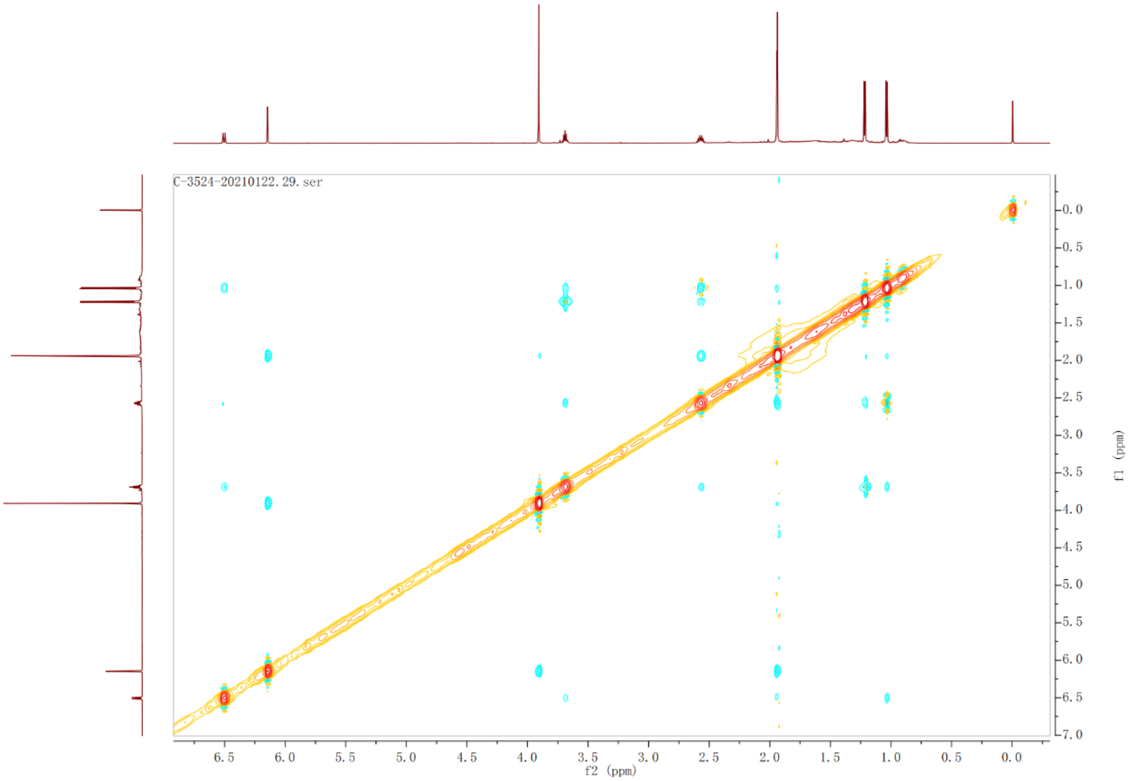

Figure S15. NOESY ($CDCl_3$) spectrum of compound **2**

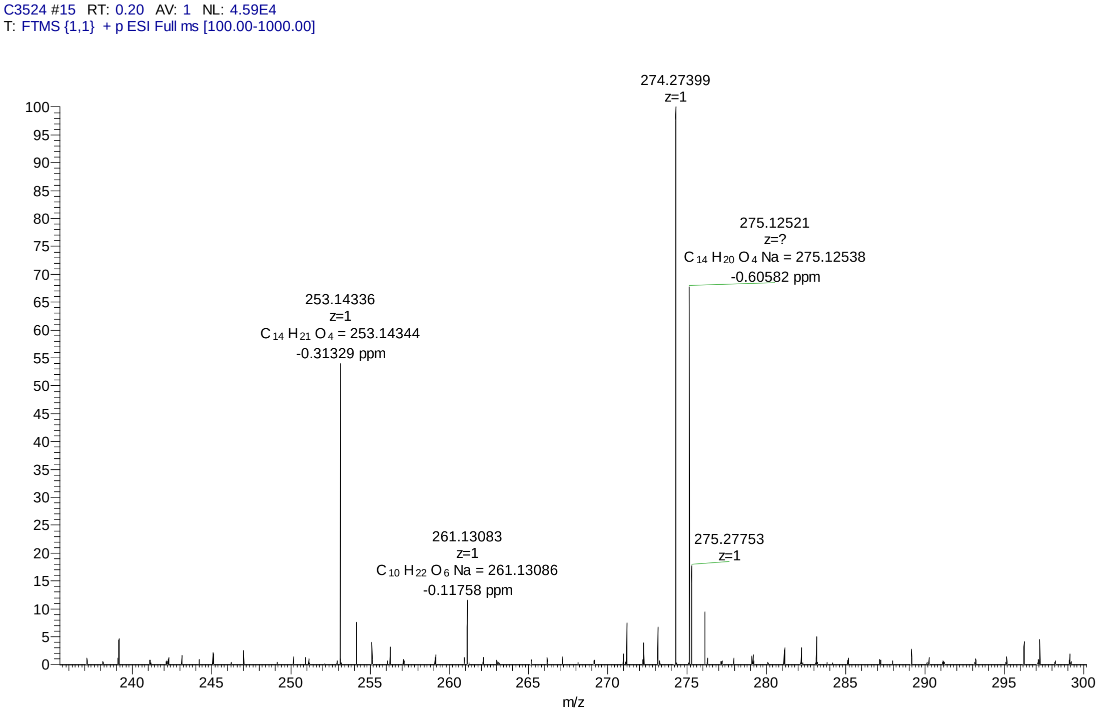

Figure S16. HR-ESI-MS spectrum of compound **2**

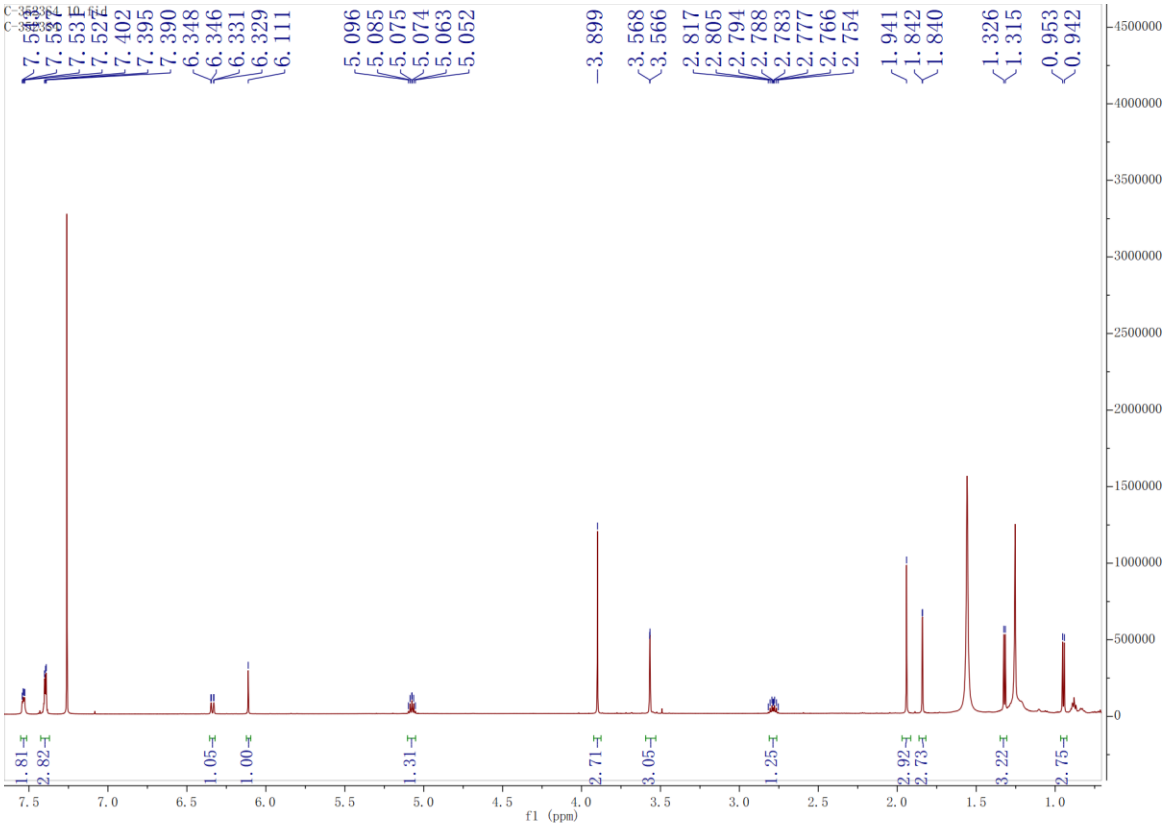

Figure S17. ^{1}H NMR (600 MHz, $CDCl_3$) spectrum of compound **1s**

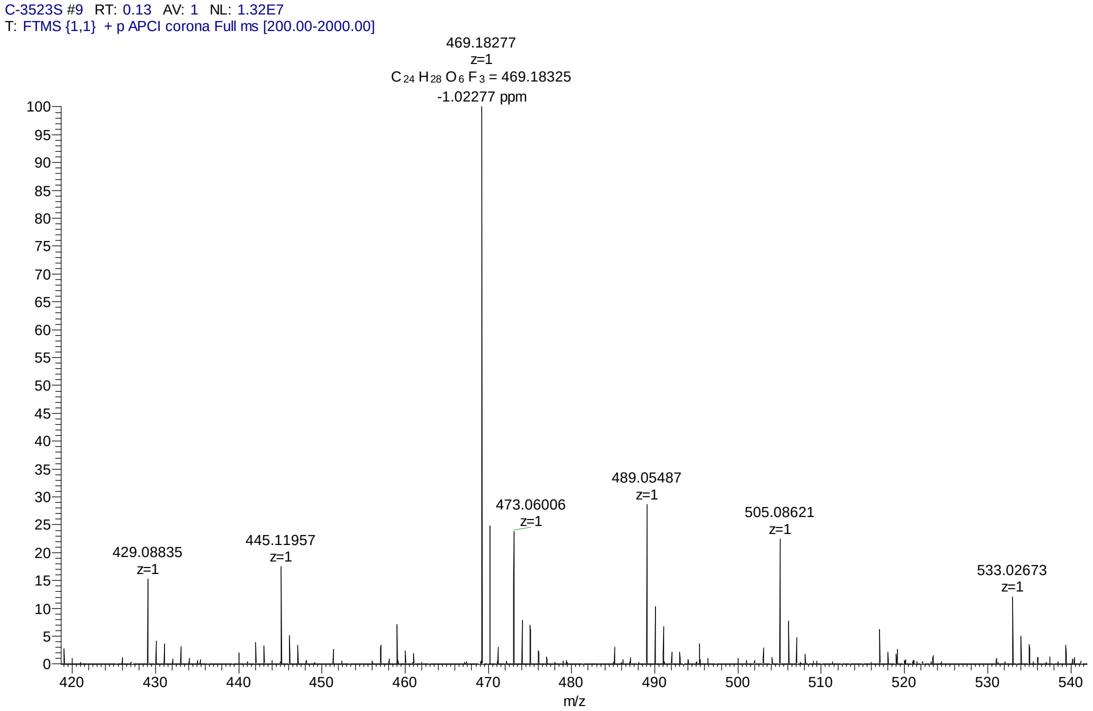

Figure S18. HR-APCI-MS spectrum of compound **1s**

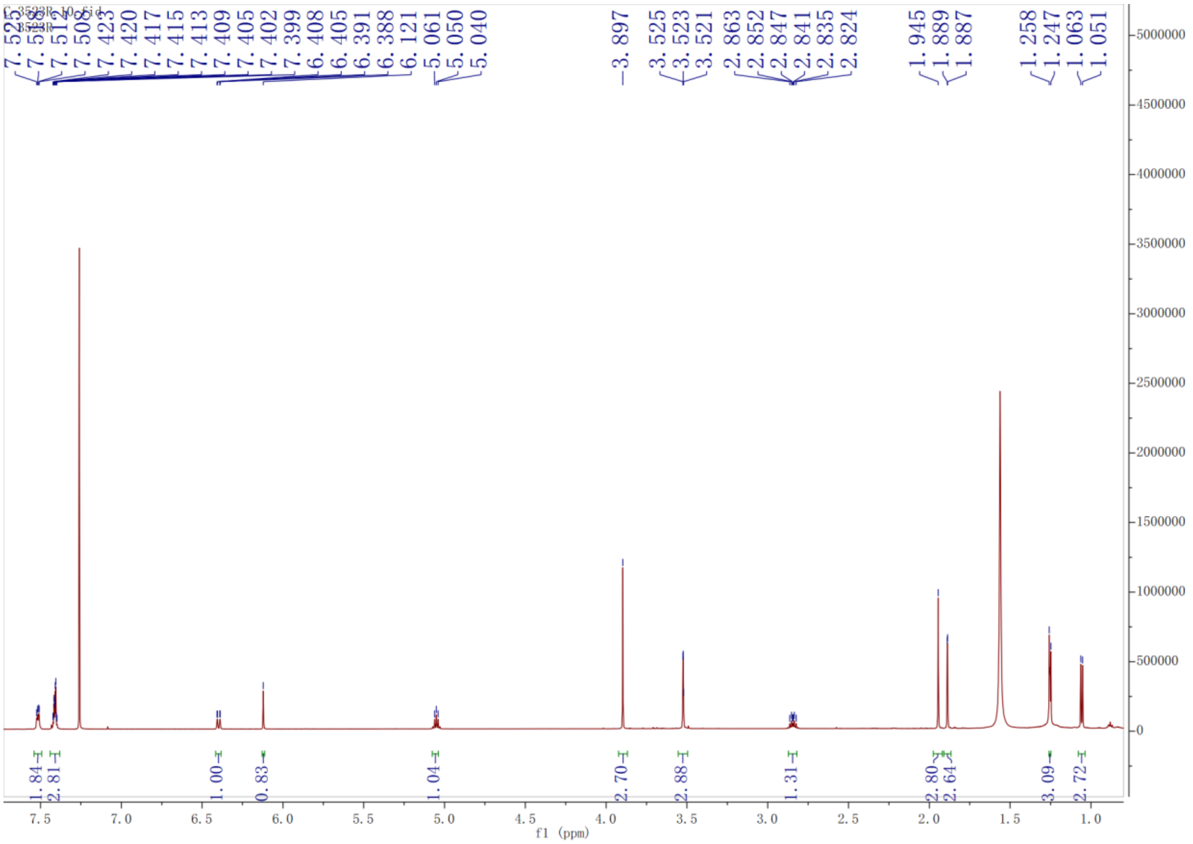

Figure S19. ^{1}H NMR (600 MHz, $CDCl_3$) spectrum of compound **1r**

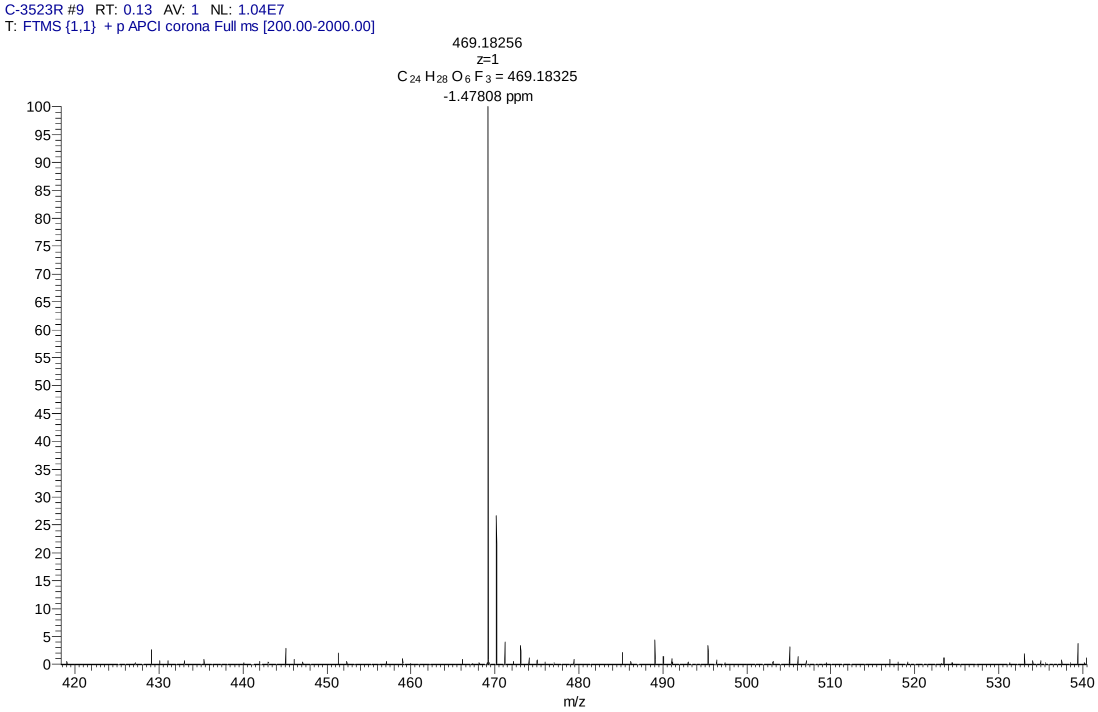

Figure S20. HR-APCI-MS spectrum of compound **1r**

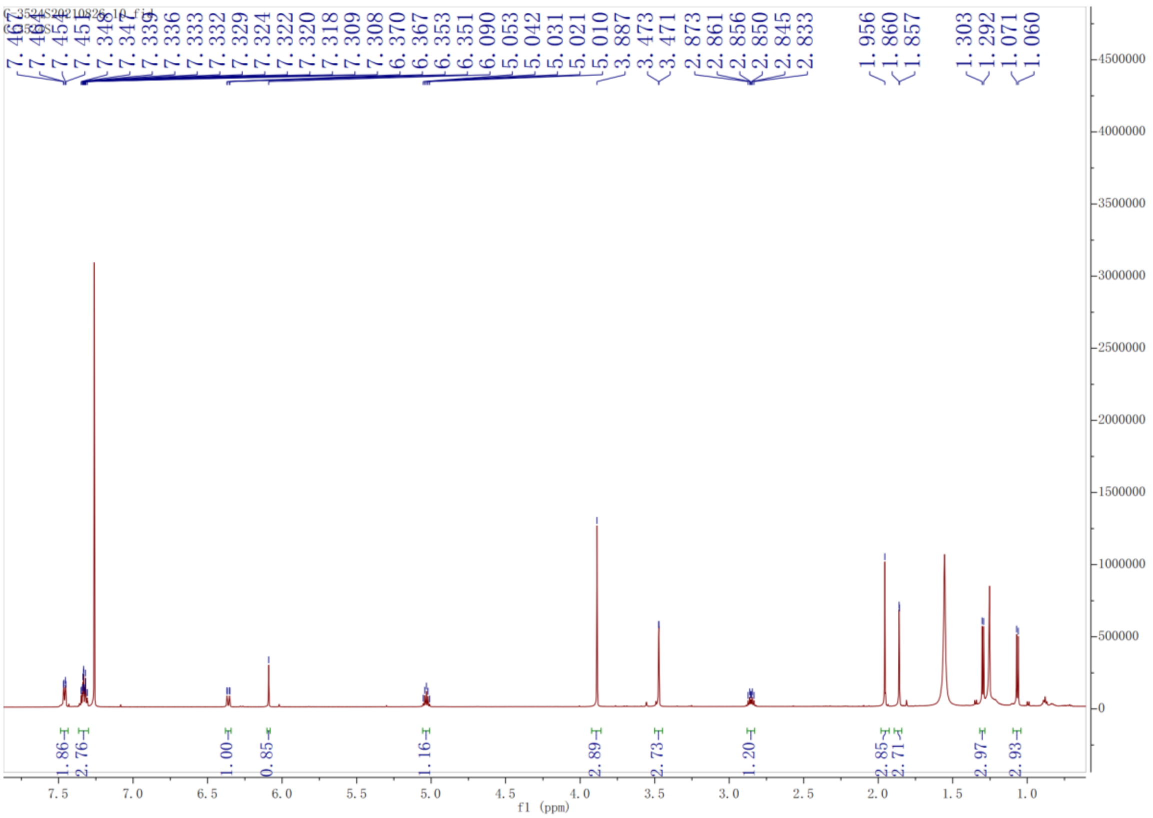

Figure S21. ^{1}H NMR (600 MHz, $CDCl_3$) spectrum of compound **2s**

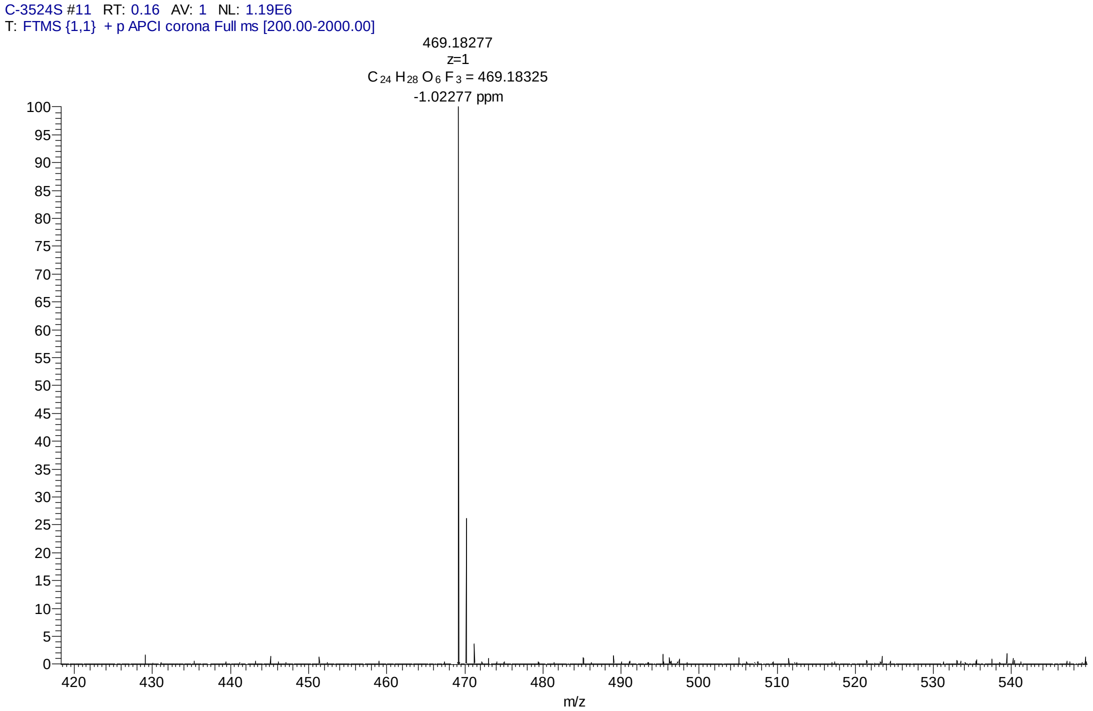

Figure S22. HR-APCI-MS spectrum of compound **2s**

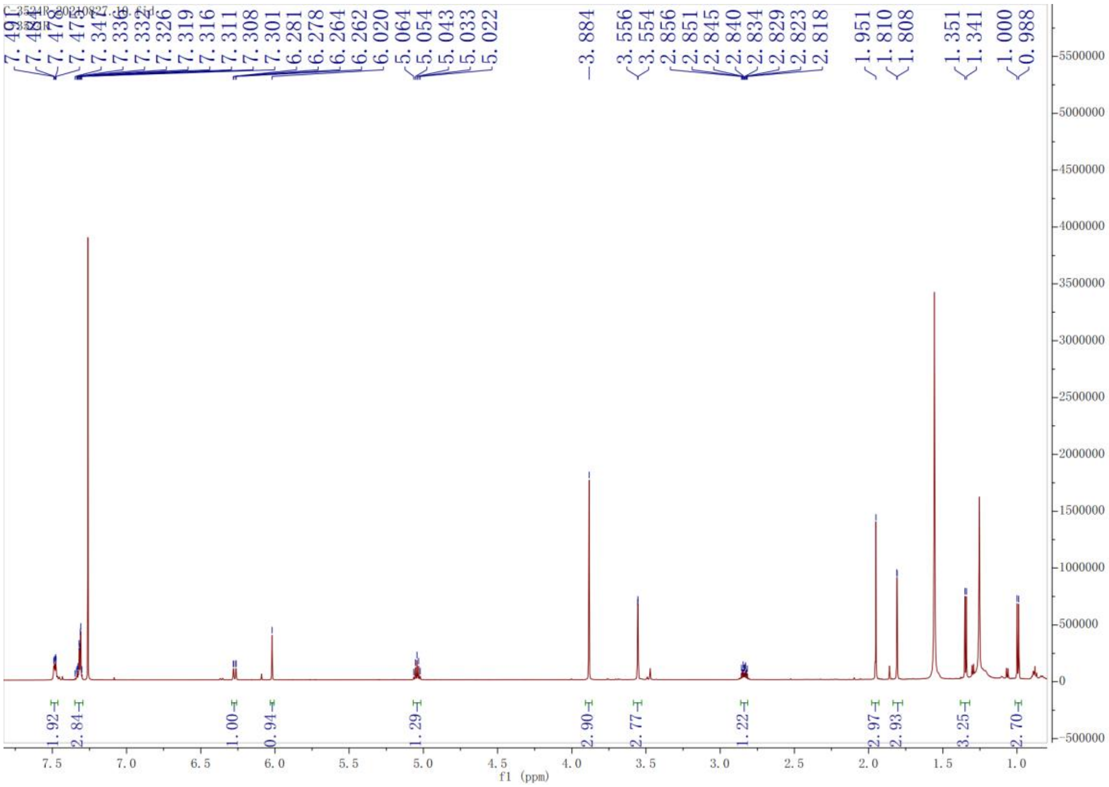

Figure S23. ^{1}H NMR (600 MHz, $CDCl_3$) spectrum of compound **2r**

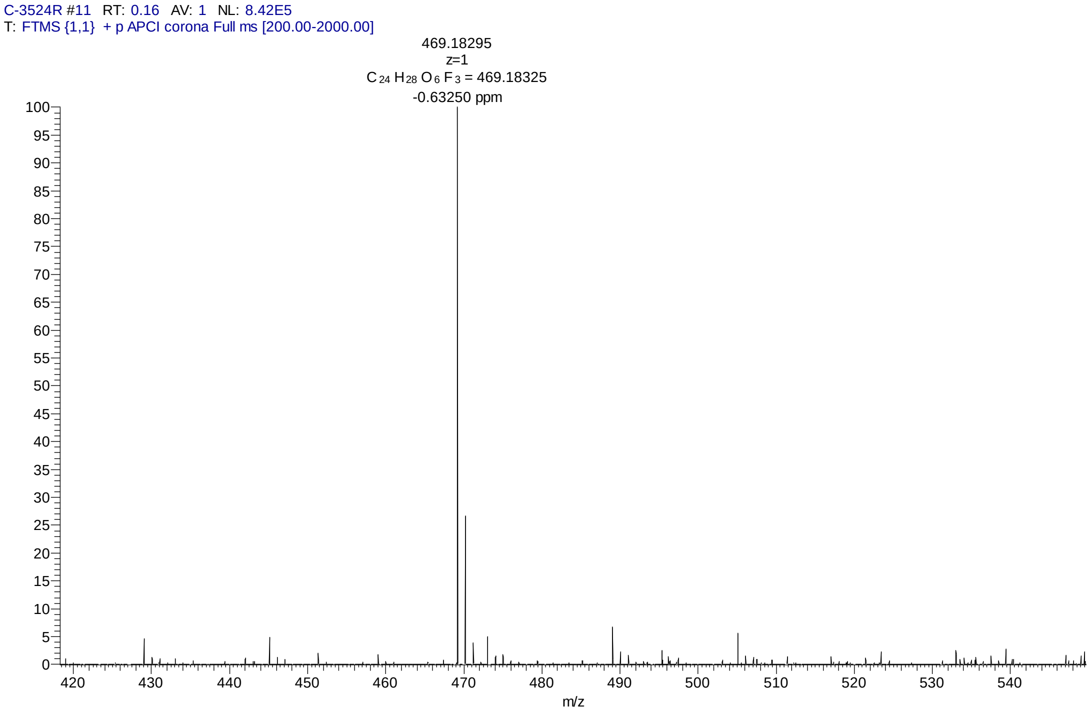

Figure S24. HR-APCI-MS spectrum of compound **2r**

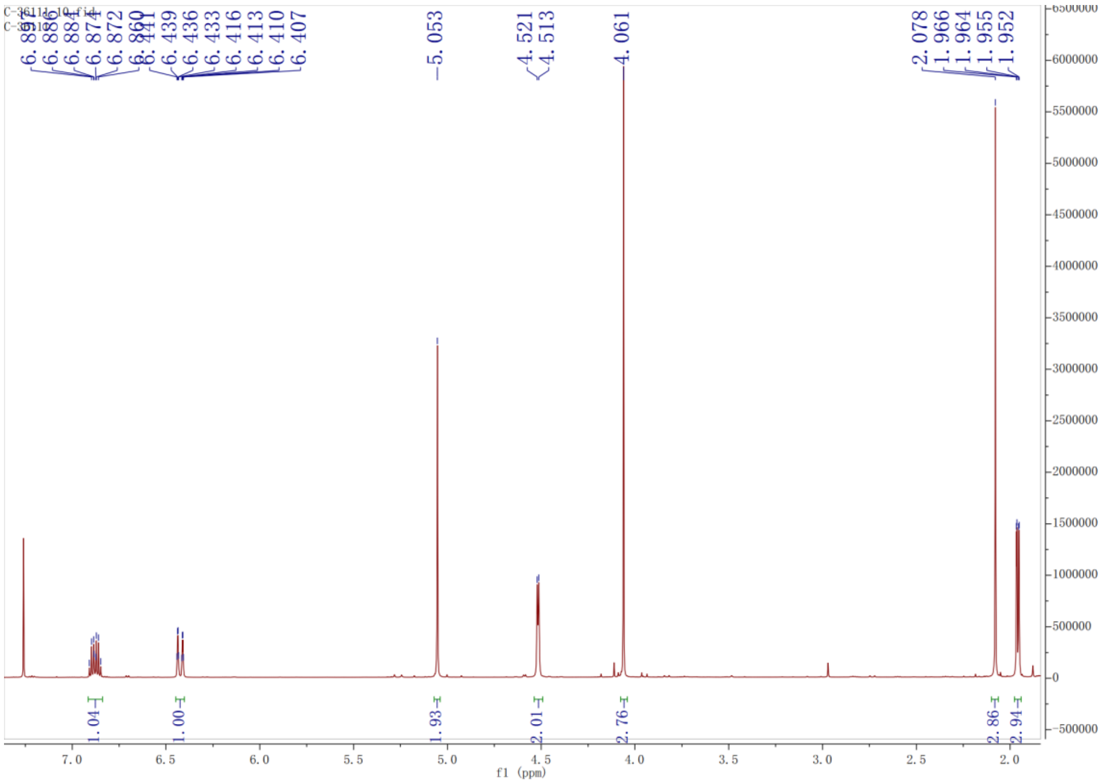

Figure S25. ^{1}H NMR (600 MHz, $CDCl_3$) spectrum of compound **3**

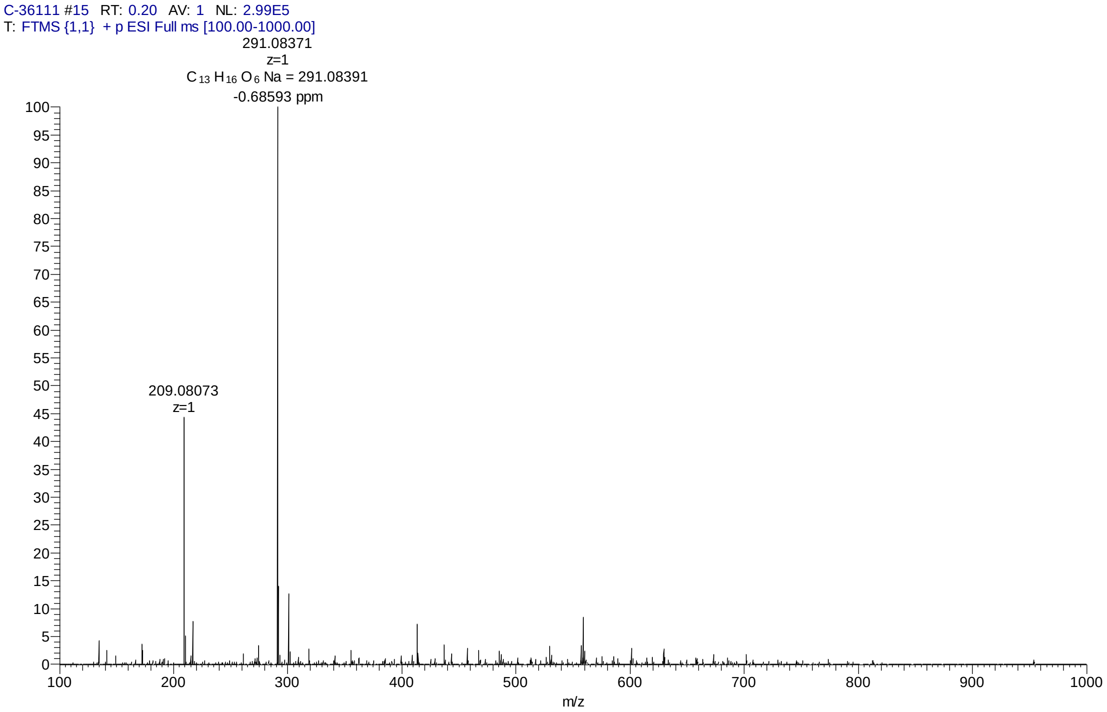

Figure S26. HR-ESI-MS spectrum of compound **3**

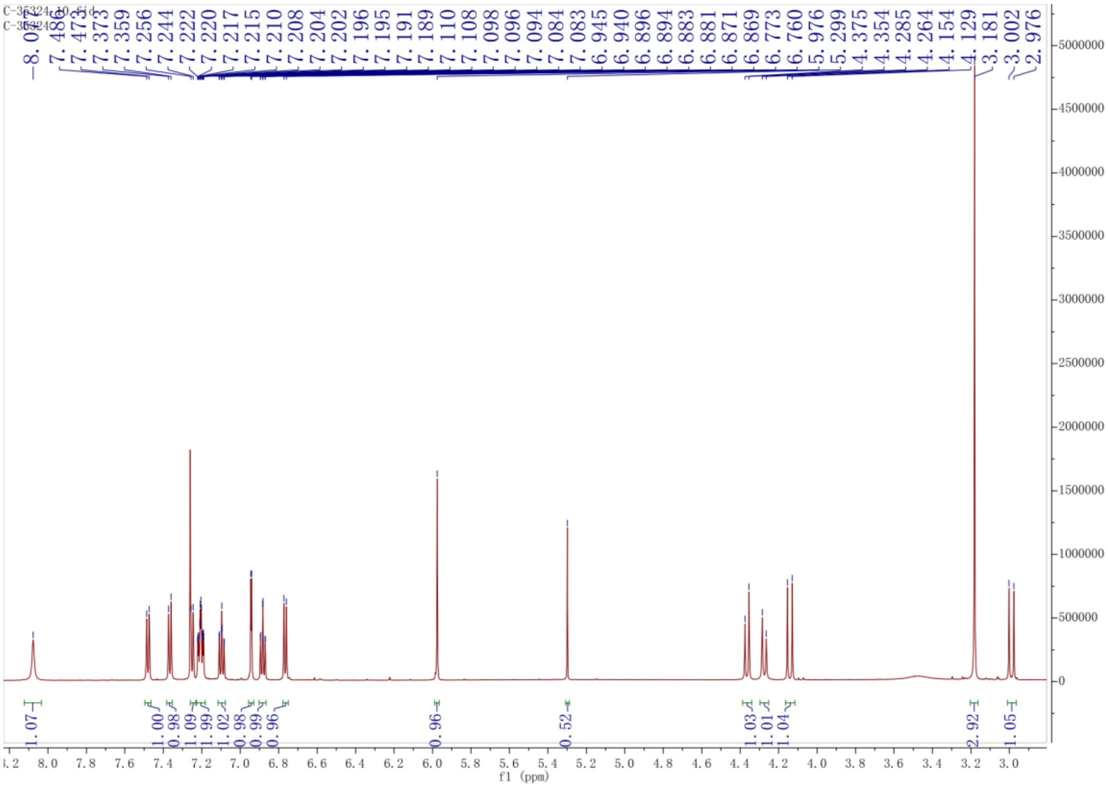

Figure S27. ^{1}H NMR (600 MHz, $CDCl_3$) spectrum of compound **4**

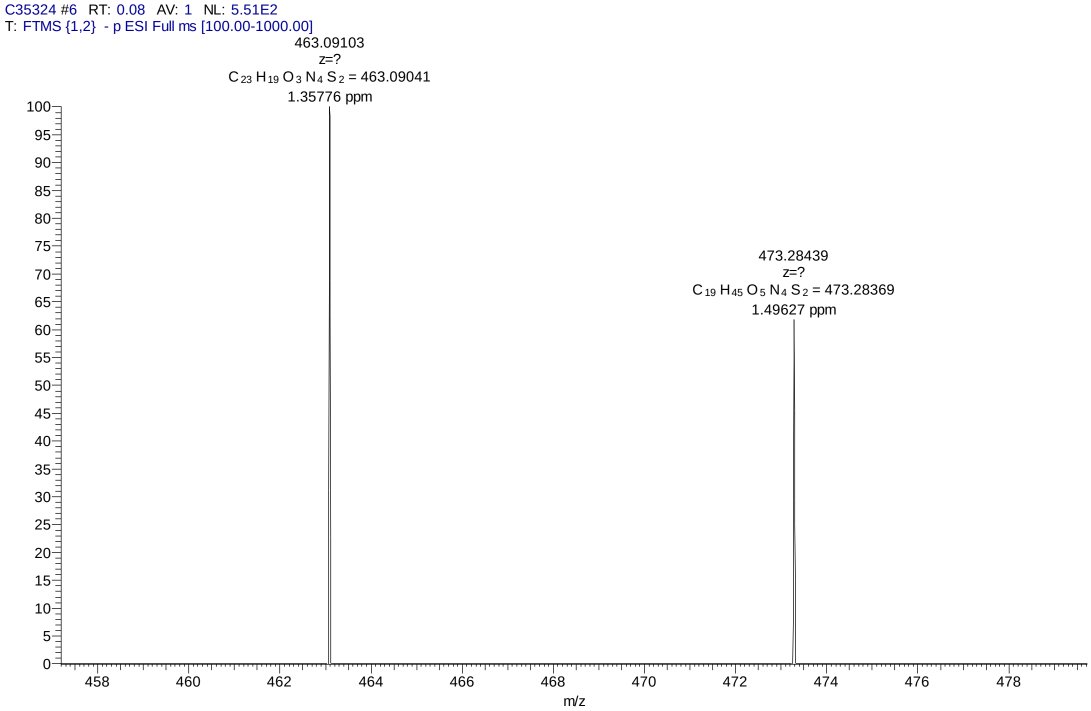

Figure S28. HR-ESI-MS spectrum of compound **4**

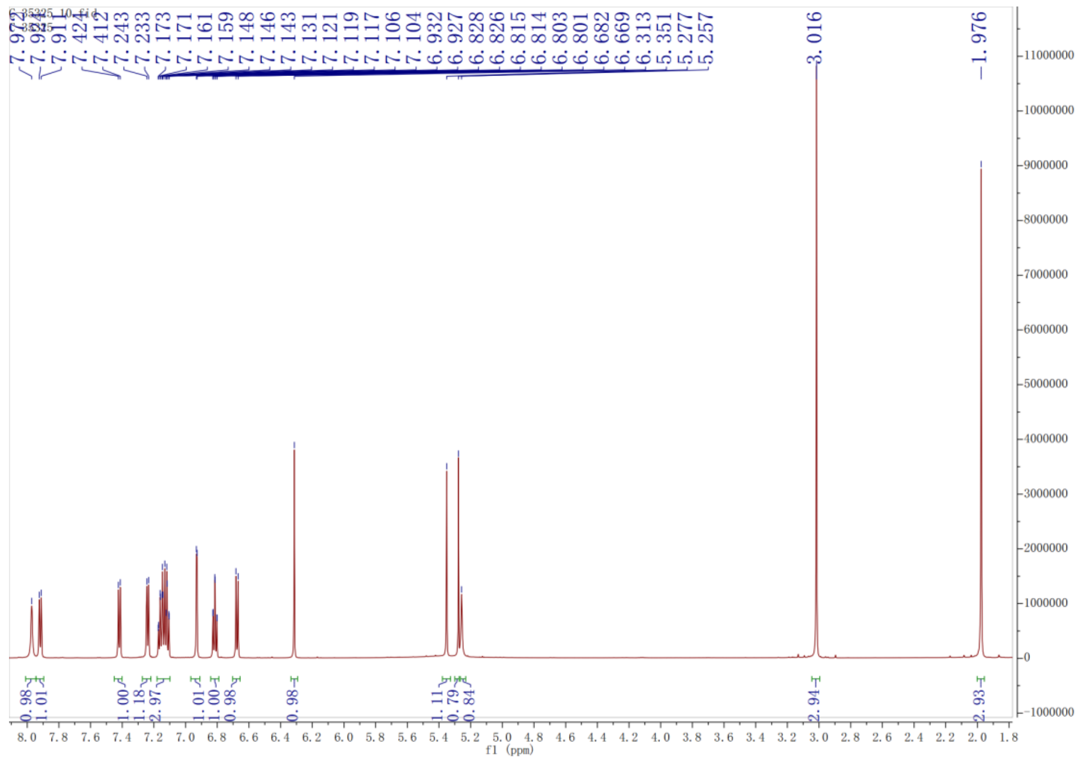

Figure S29. ^{1}H NMR (600 MHz, $CDCl_3$) spectrum of compound **5**

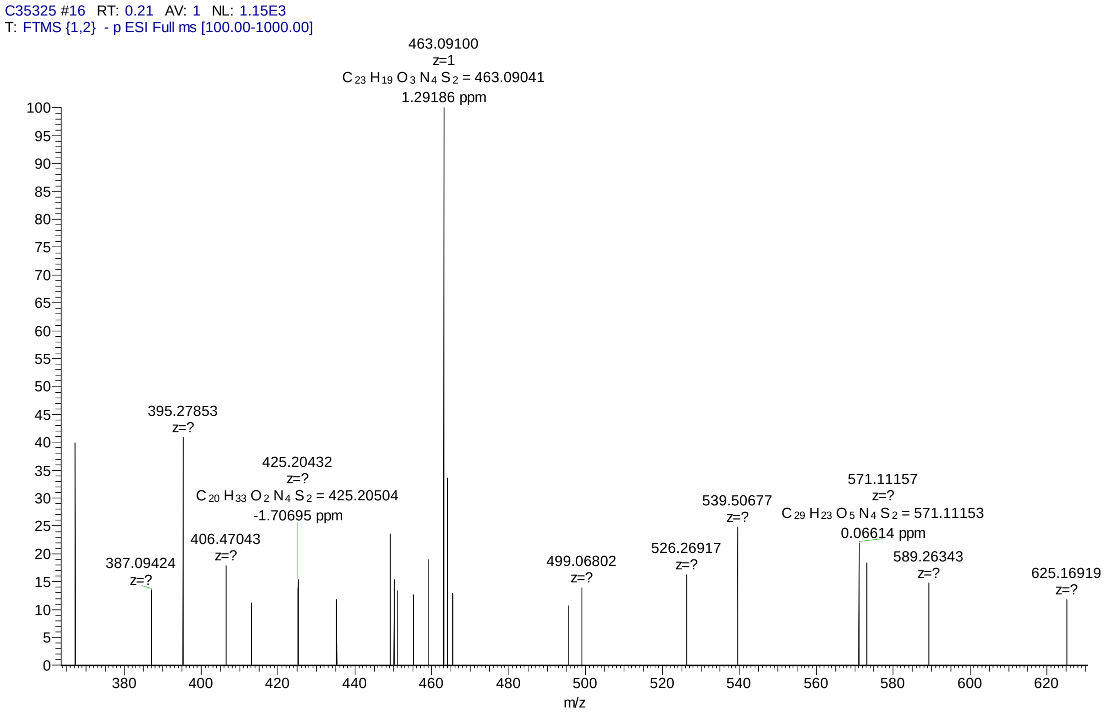

Figure S30. HR-ESI-MS spectrum of compound **5**

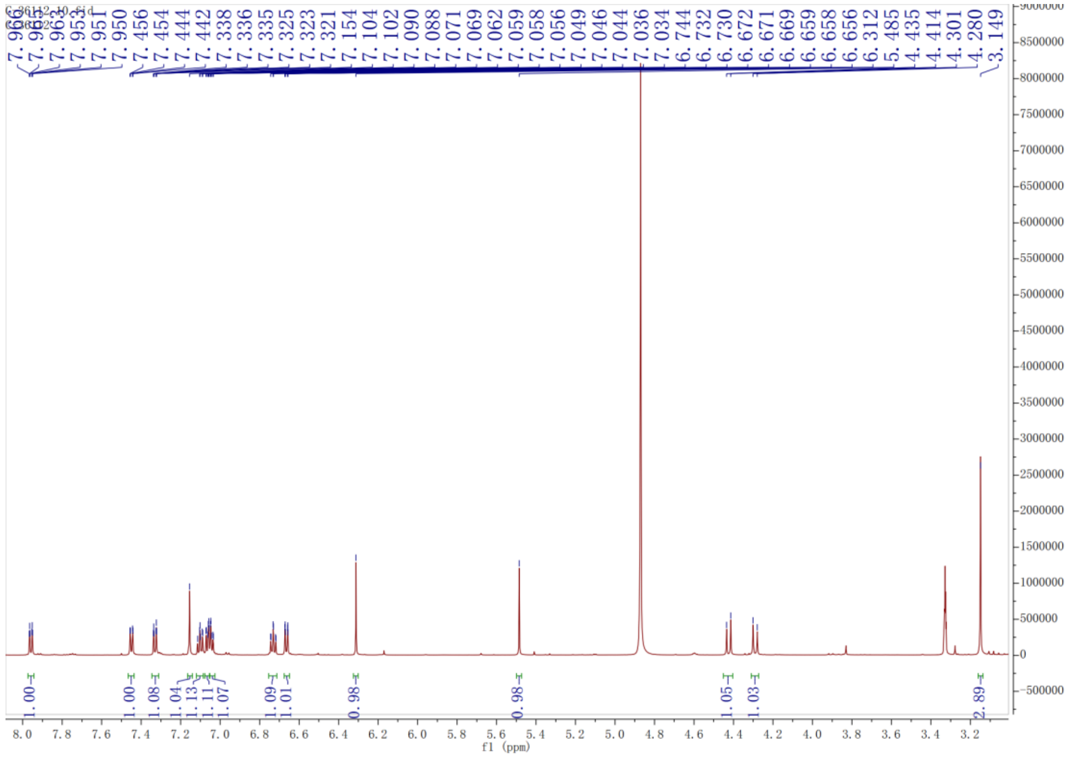

Figure S31. ^{1}H NMR (600 MHz, CD_3OD) spectrum of compound **6**

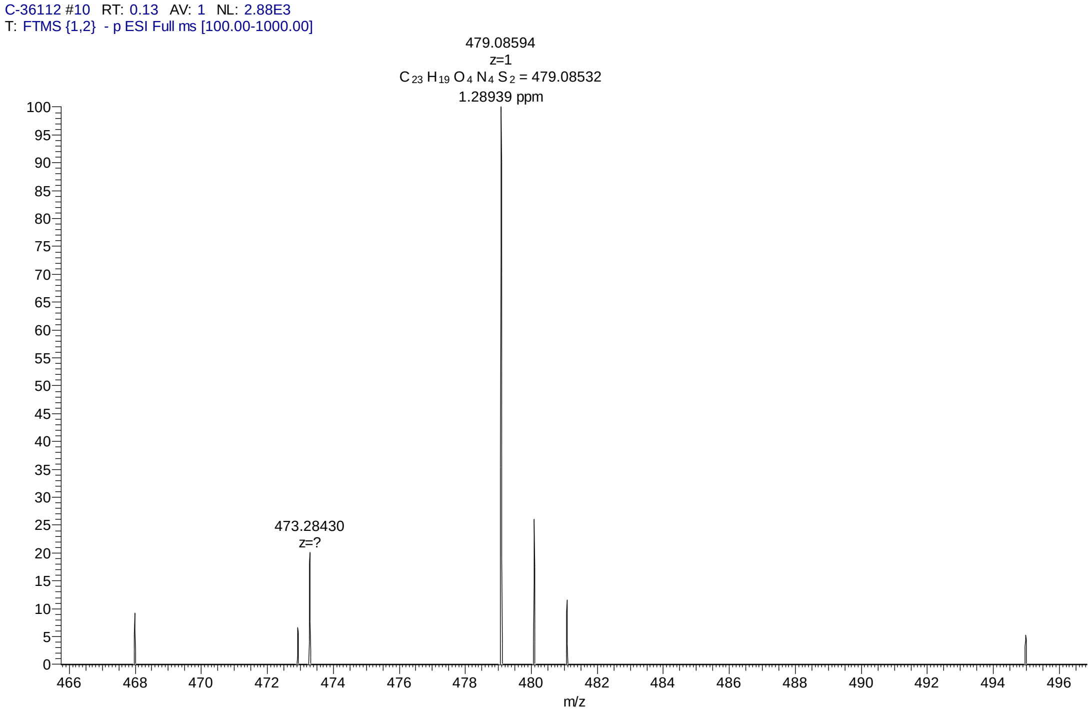

Figure S32. HR-ESI-MS spectrum of compound **6**

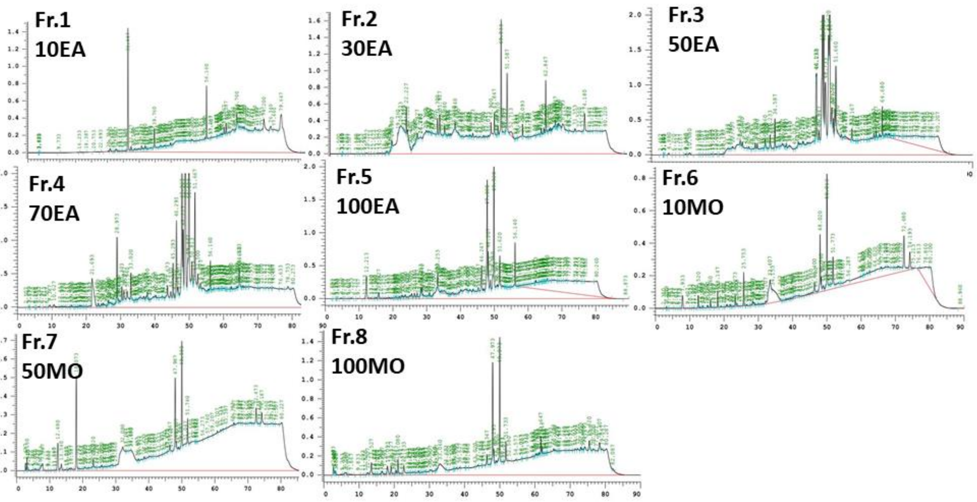

Figure S33. HPLC fingerprints of separated fractions Fr.1–Fr.8 of *A. luteoalbus* CH-6

Table S1. Identified compounds by molecular networking

Compound name	Adduct	Parent mass	CAS number	RT (min)
L-Phenylalanine, N-acetyl-	$[M+H]^+$	208.097	2018613	61.9
Triethylcitratetriethyl 2-hydroxypropane-1,2,3-tricarboxylate	$[M+H]^+$	277.128	77930	58.4
Dibutyl phthalate	$[M+H]^+$	279.159	84742	54.5
cyclo(D-Trp-L-Pro)	$[M+Na]^+$	284.139	509078493	63.5
L-Tyrosine	$[M+H]^+$	182.082	60184	6.1
CocamidopropylBetaine	$[M+H]^+$	343.296	N/A	53.4
1-Linoleoylglycerol	$[M+H]^+$	355.284	2258926	60.8
Monoolein	$[M+H]^+$	357.3	111035	51.0
Polanrazine B	$[M]^+$	377.32	394221002	59.4
Dioctyl phthalate	$[M+H]^+$	391.285	117840	63.9
6-[3-[(3,4-dimethoxyphenyl)methyl]-4-methoxy-2-(methoxymethyl)butyl]-4-methoxy-1,3-benzodioxole	$[M+H-H2O]^+$	415.211	N/A	44.5
His-Pro	$[M+H-H2O]^+$	235.122	N/A	21.9
Decaethylene glycol	$[M+H]^+$	459.28	5579668	28.2
Cyclohexasiloxane, dodecamethyl	$[M+NH_4]^+$	462.146	540976	34.2
Lyso-PC(16:0)	$[M+H]^+$	496.339	17364168	58.3
Undecaethylene glycol	$[M+H]^+$	503.311	6809707	29.4
1-(hexadecanoyloxy)-3-hydroxypropan-2-yl-octadec-9-	$[M+NH_4]^+$	612.556	29541660	50.2

enoate				
Palmitamide	$[M+H]^+$	256.263	629549	55.1
2,5-Piperazinedione, 3-(1*H*-indol-3-ylmethyl)-6-methyl-	$[M+H]^+$	258.124	17079377	24.4
cyclo(L-Tyr-L-Pro)	$[M+H]^+$	261.123	4549024	26.3
cyclo(Phe-4-Hyp)	$[M+H]^+$	261.124	N/A	26.4

Table S2. OR values of compounds **4**–**6**

Compounds	**4**	**5**	**6**
$[\alpha]_D^{20}$	+239 (0.1, $CHCl_3$)	+561 (0.1, pyridine)	+312 (0.1, MeOH)

Table S3. Antimicrobial activities of the separated fractions (50 μg/mL) of *A. luteoalbus* CH-6

Fractions	*A. salmonicida*	*C. albicans*
1	-1.23±0.40	3.52±6.20
2	75.75±0.40	66.67±0.40
3	81.02±0.60	72.18±0.25
4	80.58±0.65	70.42±0.10
5	74.43±0.35	69.95±0.30
6	76.1±1.00	71.48±0.15
7	72.26±0.10	73.47±0.40
8	72.43±0	71.58±0.30
3.1	23.12±1.11	31.56±0.32
3.2	27.59±0.20	54.93±0.40
3.3	5.89±1.35	61.5±6.40
3.4	55.1±2.35	39.52±0.05
3.5	77.33±0.70	73.83±1.15
3.6	82.67±0.72	75.52±1.15
3.7	67.98±0.40	63.31±0.82
3.8	69±0	54.55±2.15
3.5.1	72.06±0.50	66.55±0.75
3.5.2	83.22±0.15	78.64±0.60
3.5.3	78.42±0.45	75.71±0.10
3.5.4	-0.08±1.05	43.45±9.95
3.6.1	80.58±1.75	66.59±1.75
3.6.2	85.67±1.30	76.27±1.15
3.6.3	71.25±0.15	52.18±1.45
3.6.4	23.42±1.75	8.03±0.95
